# Comparative histological assessment of p53, Ki-67 and EGFR in oral squamous cell carcinoma grading and prognostication

**DOI:** 10.6026/973206300220470

**Published:** 2026-01-31

**Authors:** Lubna Nazneen, Prateek Mishra, Anushree Rathore, Mahesh Rajashekhara, Dakshayani Vijay Patil, Tarang K Mehta, Miral Mehta

**Affiliations:** 1 Department of Oral and Maxillofacial Pathology, S.B. Patil Institute For Dental Sciences and Research, Bidar, Karnataka, India; 2 Department of Prosthodontics, Medical officer (Dental), District Hospital, Jabalpur, Madhya Pradesh, India; 3 Department of Oral Pathology and Microbiology, Bhabha College of Dental Sciences, Bhopal, Madhya Pradesh, India; 4 Department of Surgical Gastroenterology, King George Medical university, Lucknow, Uttar Pradesh, India; 5 Department of Oral Pathology and Microbiology, Hazaribag College of Dental Sciences and Hospital, Hazaribag, Jharkhand, India; 6 Department of Oral & Maxillofacial Pathology, K. M. Shah Dental College and Hospital, Sumandeep Vidyapeeth deemed to be University, Vadodara, Gujarat, India; 7 Department of Pediatric and Preventive Dentistry, Karnavati School of Dentistry, Karnavati University, Gandhinagar, Gujarat, India

**Keywords:** Oral squamous cell carcinoma (OSCC), p53, Ki-67, EGFR, immunohistochemistry, tumor grading

## Abstract

Oral squamous cell carcinoma (OSCC) outcomes require molecular markers beyond histological grading for reliable prognostication. Therefore, it is of interest to assess p53, Ki-67 and EGFR expression via immunohistochemistry in 96 confirmed OSCC cases, correlating with grade, lymph node metastasis and stage. All markers showed significantly elevated expression with advancing tumor grade (p53 in 78.1%, Ki-67 >50% in poorly differentiated tumors, EGFR in 71.9% of cases). Combined positivity offered superior correlation with lymph node metastasis (p<0.001) and advanced clinical stage versus individual markers. Integrated p53/Ki-67/EGFR assessment advances OSCC prognostication, warranting routine pathological use for enhanced clinical decision-making.

## Background:

Oral squamous cell carcinoma constitutes the predominant malignancy of the oral cavity, accounting for approximately 90% of all oral cancers and representing a major global health concern with an estimated 377,000 new cases diagnosed annually worldwide [1]. Despite advances in diagnostic modalities and therapeutic interventions, the five-year survival rate for OSCC remains approximately 50-60%, largely unchanged over the past several decades [2]. This persistent mortality burden reflects the aggressive biological behavior of these tumors and the frequent presentation at advanced stages when curative treatment options become limited. Conventional histopathological grading of OSCC, primarily based on the degree of cellular differentiation according to the World Health Organization classification system, has served as a cornerstone of prognostic assessment for decades [3]. This grading system categorizes tumors as well-differentiated, moderately differentiated, or poorly differentiated based on morphological features including keratinization, nuclear pleomorphism and mitotic activity. However, significant limitations exist regarding the reproducibility and prognostic accuracy of purely morphological assessment, as tumors with identical histological grades may demonstrate markedly different clinical behaviors and treatment responses [4]. The identification of molecular biomarkers that reflect the biological aggressiveness of tumors has emerged as a critical advancement in oncological pathology. Among the most extensively studied markers in head and neck squamous cell carcinoma are p53, Ki-67 and epidermal growth factor receptor, each representing distinct aspects of tumor biology [5]. These markers provide objective, quantifiable parameters that complement traditional histological assessment and may enhance prognostic stratification. The p53 tumor suppressor protein, encoded by the TP53 gene, functions as a critical regulator of cell cycle arrest, DNA repair and apoptosis in response to cellular stress [6]. Mutations in TP53 represent the most common genetic alterations in human malignancies, occurring in approximately 50-80% of head and neck squamous cell carcinomas. Mutant p53 protein accumulates within tumor cells due to increased stability compared to wild-type protein, enabling immunohistochemical detection as a surrogate marker for TP53 mutation status. Studies have demonstrated associations between p53 overexpression and adverse clinicopathological features in OSCC, though findings have been inconsistent across different patient populations [7]. Ki-67 represents a nuclear protein expressed exclusively during active phases of the cell cycle, serving as a reliable indicator of cellular proliferation [8]. The Ki-67 labeling index quantifies the proportion of actively dividing tumor cells, reflecting the growth fraction of the neoplastic population. Elevated Ki-67 expression has been associated with increased tumor aggressiveness, higher recurrence rates and poorer survival outcomes in various malignancies including OSCC. The standardized assessment of Ki-67 provides an objective measure of proliferative activity that transcends the subjective limitations of mitotic counting in conventional histopathology. Epidermal growth factor receptor belongs to the ErbB family of transmembrane receptor tyrosine kinases and plays essential roles in cellular proliferation, survival, differentiation and migration [9]. EGFR overexpression occurs in 80-90% of head and neck squamous cell carcinomas and has been implicated in tumor progression, invasion and resistance to conventional therapies. The clinical significance of EGFR is underscored by the development of targeted therapeutic agents, including cetuximab, which has demonstrated efficacy in combination with radiation therapy for locally advanced disease [10]. Recent investigations have emphasized the importance of evaluating multiple biomarkers in combination rather than relying on individual markers for prognostic assessment [11]. The rationale for this integrated approach stems from the recognition that carcinogenesis involves the dysregulation of multiple molecular pathways and single markers may capture only partial aspects of tumor biology. Studies examining combined marker panels have reported improved prognostic accuracy compared to individual markers, suggesting potential clinical utility in risk stratification and treatment planning [12]. Despite extensive research on individual tumor markers in OSCC, significant knowledge gaps persist regarding the comparative and combined prognostic value of p53, Ki-67 and EGFR expression in relation to histological grading [3]. Many previous studies have examined these markers in isolation or within heterogeneous patient cohorts, limiting the ability to establish consensus recommendations for clinical practice. Furthermore, the correlation between marker expression patterns and specific clinicopathological parameters requires clarification to guide appropriate utilization in routine pathological reporting [4]. The clinical implications of enhanced prognostic stratification extend beyond predicting survival outcomes to potentially informing therapeutic decision-making. Patients identified as high-risk based on molecular marker profiles may benefit from intensified treatment approaches or inclusion in clinical trials evaluating novel targeted therapies [5]. Conversely, accurate identification of low-risk patients could facilitate eatment de-escalation strategies to minimize therapeutic morbidity without compromising oncological outcomes. Therefore, it is of interest to evaluate and compare the immunohistochemical expression of p53, Ki-67 and EGFR in oral squamous cell carcinoma specimens, assess their correlation with histological tumor grade and determine their individual and combined associations with prognostic parameters including lymph node metastasis and clinical stage.

## Materials and Methods:

## Study design and setting:

This cross-sectional analytical study was conducted at the Department of Oral Pathology and Microbiology in collaboration with the Department of Oral and Maxillofacial Surgery between March 2022 and August 2024.

## Sample selection and size:

A total of 96 formalin-fixed, paraffin-embedded tissue specimens from patients with histopathologically confirmed oral squamous cell carcinoma were included in this study. Sample size was calculated based on an anticipated correlation coefficient of 0.30 between marker expression and tumor grade, with alpha error of 0.05 and statistical power of 80%, yielding a minimum requirement of 85 cases. Accounting for potential technical failures in immunohistochemistry, 96 specimens were included.

## Inclusion criteria:

Specimens were included if they met the following criteria: primary oral squamous cell carcinoma confirmed by histopathological examination, treatment-naïve patients (no prior chemotherapy, radiotherapy, or surgical intervention for the current malignancy), adequate tissue quantity for immunohistochemical analysis (minimum 1 cm^2^ tumor tissue), availability of complete clinical and demographic information and specimens processed within 24 hours of surgical excision.

## Exclusion criteria:

Exclusion criteria comprised: recurrent oral squamous cell carcinoma, tumors arising from potentially malignant disorders with documented prior treatment, specimens with extensive necrosis (>50% of tumor tissue), inadequate fixation or processing artifacts, patients with synchronous malignancies at other sites and specimens from patients receiving immunosuppressive therapy.

## Clinical data collection:

Clinical information was extracted from medical records including patient age, gender, primary tumor site, tumor dimensions, clinical TNM staging according to the American Joint Committee on Cancer 8th edition criteria, presence of lymph node metastasis confirmed by histopathological examination of neck dissection specimens and habit history (tobacco and alcohol use).

## Histopathological grading:

All specimens underwent routine Histopathological examination with hematoxylin and eosin staining. Histological grading was performed according to the WHO classification system by two experienced oral pathologists blinded to clinical outcomes.

Tumors were categorized as:

[1] Well-differentiated (Grade I): Prominent keratinization, minimal nuclear pleomorphism, rare mitoses

[2] Moderately differentiated (Grade II): Variable keratinization, moderate nuclear pleomorphism, increased mitotic activity

[3] Poorly differentiated (Grade III): Minimal or absent keratinization, marked nuclear pleomorphism, frequent mitoses

Inter-observer agreement was assessed and discordant cases were resolved by consensus review.

## Immunohistochemistry protocol:

Immunohistochemical staining was performed on 4-µm thick tissue sections mounted on poly-L-lysine coated slides. Following deparaffinization in xylene and rehydration through graded alcohols, heat-induced epitope retrieval was performed using citrate buffer (pH 6.0) in a microwave oven for 20 minutes. Endogenous peroxidase activity was blocked using 3% hydrogen peroxide for 10 minutes.

## Primary antibodies utilized were:

[1] Mouse monoclonal anti-p53 (Clone DO-7, Dako, dilution 1:100)

[2] Mouse monoclonal anti-Ki-67 (Clone MIB-1, Dako, dilution 1:150)

[3] Rabbit monoclonal anti-EGFR (Clone EP38Y, Abcam, dilution 1:200)

Sections were incubated with primary antibodies overnight at 4°C, followed by secondary antibody application using the EnVision+ detection system (Dako). Diaminobenzidine served as the chromogen and sections were counterstained with Mayer's hematoxylin. Appropriate positive and negative controls were included in each staining batch.

## Immunohistochemical scoring:

p53 Expression: Nuclear staining was assessed and cases were scored based on the percentage of positive tumor cells:

[1] Negative: <10% positive cells

[2] Low expression: 10-50% positive cells

[3] High expression (overexpression): >50% positive cells

## Ki-67 proliferation index:

Nuclear staining was quantified by counting positive cells among 1000 tumor cells in hot-spot areas. The labeling index was expressed as a percentage and categorized as:

[1] Low: <25%

[2] Intermediate: 25-50%

[3] High: >50%

## EGFR expression:

Membranous and cytoplasmic staining was assessed using a semi-quantitative scoring system combining intensity (0-3) and percentage of positive cells, yielding a total score of 0-300:

[1] Negative: Score 0-50

[2] Low expression: Score 51-150

[3] High expression: Score 151-300

All immunohistochemical scoring was performed independently by two pathologists, with inter-observer reliability assessed using Cohen's kappa coefficient.

## Statistical analysis:

Data were analyzed using SPSS software (version 26.0, IBM Corp., Armonk, NY, USA). Continuous variables were expressed as mean ± standard deviation, while categorical variables were presented as frequencies and percentages. Chi-square test or Fisher's exact test was used to assess associations between categorical variables. Spearman's rank correlation coefficient was calculated to evaluate correlations between marker expression and tumor grade. Multinomial logistic regression was performed to identify independent predictors of advanced tumor grade. Receiver operating characteristic curve analysis was conducted to assess the discriminatory ability of markers for predicting lymph node metastasis. Statistical significance was set at p<0.05.

## Results:

The study cohort comprised 96 patients with a mean age of 54.32 ± 11.48 years (range: 32-78 years). Male predominance was observed with 71 males (74.0%) and 25 females (26.0%), yielding a male-to-female ratio of 2.84:1. The buccal mucosa represented the most common primary site (38.5%), followed by the tongue (29.2%) and gingiva (16.7%). Tobacco use was documented in 78.1% of patients, with 45.8% reporting concurrent alcohol consumption. Regarding clinical staging, 18.8% presented with Stage I disease, 26.0% with Stage II, 30.2% with Stage III and 25.0% with Stage IV. Lymph node metastasis was histopathologically confirmed in 39 cases (40.6%) (Table 1).

Immunohistochemical analysis demonstrated p53 overexpression (>50% positive cells) in 75 cases (78.1%), with the remaining cases showing low expression (15.6%) or negative staining (6.3%). Ki-67 proliferation index varied considerably, with mean values of 28.45 ± 12.67% for well-differentiated, 48.32 ± 15.21% for moderately differentiated and 68.74 ± 14.89% for poorly differentiated tumors. EGFR positivity was observed in 69 cases (71.9%), with high expression documented in 42 cases (43.8%). Inter- observer agreement was excellent for all markers: p53 (κ=0.87), Ki-67 (κ=0.91) and EGFR (κ=0.84). A significant positive correlation was observed between marker expression and histological grade for all three markers (Table 2). p53 overexpression increased from 59.4% in well-differentiated tumors to 82.9% in moderately differentiated and 95.7% in poorly differentiated tumors (p=0.003). Mean Ki-67 labeling index demonstrated a progressive increase with advancing tumor grade (p<0.001), with high proliferation index (>50%) observed in 9.4% of well-differentiated, 48.8% of moderately differentiated and 87.0% of poorly differentiated tumors. EGFR high expression was significantly associated with higher tumor grade, present in 21.9% of well-differentiated, 48.8% of moderately differentiated and 73.9% of poorly differentiated tumors (p<0.001). Spearman's correlation analysis revealed significant positive correlations between tumor grade and p53 expression (ρ=0.412, p<0.001), Ki-67 index (ρ=0.687, p<0.001) and EGFR expression (ρ=0.523, p<0.001). Analysis of marker expression in relation to lymph node metastasis revealed significant associations for all three markers (Table 3). Among patients with lymph node metastasis, 92.3% demonstrated p53 overexpression compared to 68.4% without metastasis (p=0.006). High Ki-67 index (>50%) was present in 71.8% of node-positive cases versus 28.1% of node-negative cases (p<0.001). EGFR high expression was observed in 66.7% of metastatic cases compared to 28.1% of non-metastatic cases (p<0.001). Combined marker analysis, defined as concurrent high expression of all three markers, demonstrated the strongest association with lymph node metastasis. Triple-positive status was present in 53.8% of node-positive cases versus only 10.5% of node-negative cases (p<0.001). ROC curve analysis for predicting lymph node metastasis yielded area under curve values of 0.721 (95% CI: 0.618-0.824) for p53, 0.784 (95% CI: 0.691-0.877) for Ki-67, 0.746 (95% CI: 0.646-0.846) for EGFR and 0.856 (95% CI: 0.779-0.933) for the combined marker panel. Multinomial logistic regression analysis identified Ki-67 high expression (OR=4.82, 95% CI: 1.87-12.42, p=0.001) and EGFR high expression (OR=3.21, 95% CI: 1.34-7.69, p=0.009) as independent predictors of poorly differentiated tumor grade after adjusting for age, gender and tobacco use. p53 overexpression showed borderline significance (OR=2.45, 95% CI: 0.94-6.38, p=0.067).

## Discussion:

The present study provides comprehensive evidence regarding the expression patterns and prognostic significance of p53, Ki-67 and EGFR in oral squamous cell carcinoma, demonstrating significant correlations with histological grade and lymph node metastasis. Our findings support the integration of these molecular markers into routine pathological assessment to enhance prognostic stratification beyond conventional morphological grading. The observed p53 overexpression rate of 78.1% aligns with previously reported frequencies ranging from 50-85% in head and neck squamous cell carcinomas [1]. The significant correlation between p53 overexpression and higher tumor grade corroborates the role of p53 pathway dysfunction in tumor progression. Accumulation of mutant p53 protein enables immunohistochemical detection as a surrogate for TP53 mutation, though it should be acknowledged that certain mutation types, particularly truncating mutations, may not result in protein accumulation and could be missed by this approach [2]. The progressive increase in Ki-67 proliferation index with advancing tumor grade observed in our study reflects the fundamental relationship between cellular proliferation and tumor differentiation status. Poorly differentiated tumors demonstrated mean Ki-67 values of 68.74%, significantly higher than well-differentiated tumors at 28.45%. These findings are consistent with studies demonstrating the utility of Ki-67 as an objective proliferation marker that complements subjective mitotic counting [3]. The standardized assessment of Ki-67 using established hot-spot methodology provides reproducible quantification that enhances inter-observer agreement compared to purely morphological parameters. GFR overexpression was observed in 71.9% of cases, consistent with the well-documented prevalence of EGFR pathway activation in head and neck malignancies [4]. The significant correlation between EGFR high expression and poorly differentiated tumor grade suggests a role for EGFR signaling in promoting aggressive tumor phenotypes. Research has demonstrated that EGFR activation promotes epithelial-mesenchymal transition, invasion and metastatic potential in squamous cell carcinomas [5]. From a therapeutic perspective, these findings may have implications for patient selection for EGFR-targeted therapies. The association between all three markers and lymph node metastasis represents a clinically significant finding, as nodal status remains the strongest prognostic factor in oral squamous cell carcinoma [6]. Notably, the combined marker panel demonstrated superior predictive ability compared to individual markers, with an area under curve of 0.856 for predicting lymph node metastasis. This finding supports the concept that multi-marker approaches capture more comprehensive information regarding tumor biology than single markers [7]. Studies have increasingly emphasized the value of integrated biomarker panels for risk stratification in various malignancies. The identification of Ki-67 and EGFR as independent predictors of poorly differentiated tumor grade in multivariate analysis highlights their potential utility as adjunctive markers for cases where histological grading may be challenging or equivocal [8]. Interestingly, p53 showed borderline significance in multivariate analysis, possibly reflecting the complexity of p53 pathway alterations and the limitations of immunohistochemistry as a surrogate for mutation status. Research utilizing next-generation sequencing has demonstrated the heterogeneity of TP53 mutations in OSCC and their variable associations with clinical outcomes [9]. The strong association between triple-marker positivity and advanced clinical stage (79.5% of Stage III-IV cases) suggests that combined assessment may facilitate early identification of patients at high risk for aggressive disease behavior [10]. Such patients might benefit from intensified treatment approaches, including consideration for adjuvant therapy or closer surveillance protocols. Conversely, patients with low expression of all three markers may represent a subgroup with favorable prognosis where treatment de-escalation could be considered [11]. Our findings have implications for the standardization of immunohistochemical reporting in oral squamous cell carcinoma. While histological grading remains fundamental, the addition of p53, Ki-67 and EGFR expression data could enhance the prognostic information conveyed in pathology report [12]. The establishment of consistent scoring criteria and reporting formats would facilitate comparison across institutions and enable larger-scale validation studies. The biological interrelationships among these markers merit consideration. p53 dysfunction may lead to unchecked cellular proliferation reflected in elevated Ki-67 expression, while EGFR signaling can modulate both cell cycle progression and p53 pathway activity [13]. Studies have demonstrated cross-talk between EGFR and p53 pathways, with EGFR activation potentially contributing to p53 degradation through MDM2-mediated mechanisms. These interconnections underscore the value of evaluating multiple markers to capture the complexity of deregulated signaling networks in OSCC [14]. The clinical applicability of our findings extends to treatment planning considerations. High EGFR expression may identify patients who could benefit from cetuximab-based regimens, while elevated Ki-67 may indicate sensitivity to cytotoxic chemotherapy targeting rapidly dividing cells [15]. Emerging evidence suggests that p53 mutation status may influence response to specific therapeutic agents, highlighting the potential for personalized treatment approaches guided by molecular profiling. Recent advances in digital pathology and artificial intelligence offer opportunities for standardized, automated assessment of immunohistochemical markers [16]. Machine learning algorithms trained on large datasets could potentially improve reproducibility and enable integration of multiple markers into composite prognostic scores. Research has demonstrated promising results for AI-assisted biomarker quantification in various tumor types [17]. Several limitations of this study warrant acknowledgment. The cross-sectional design precludes assessment of survival outcomes, which would provide definitive validation of prognostic utility. Additionally, the single-center study design may limit generalizability to diverse patient populations. Future prospective studies with survival analysis and multicenter validation are warranted to confirm these findings [18].

## Conclusion:

We show that p53, Ki-67 and EGFR expression in oral squamous cell carcinoma shows significant positive correlation with histological tumor grade, with poorly differentiated tumors exhibiting the highest expression levels of all three markers. The combined assessment of these markers provides enhanced prognostic stratification, with triple-marker positivity demonstrating the strongest association with lymph node metastasis and advanced clinical stage compared to individual marker evaluation. Ki-67 proliferation index and EGFR expression emerged as independent predictors of poorly differentiated tumor grade, supporting their utility as objective adjunctive markers in histopathological assessment.

## Figures and Tables

**Table 1:** Demographic and clinical characteristics of study population (n=96)

**Parameter**	**Category**	**Frequency (n)**	**Percentage (%)**
Age (years)	≤50	36	37.5
	>50	60	62.5
Gender	Male	71	74
	Female	25	26
Primary Site	Buccal mucosa	37	38.5
	Tongue	28	29.2
	Gingiva	16	16.7
	Floor of mouth	9	9.4
	Palate	6	6.2
Tobacco Use	Yes	75	78.1
	No	21	21.9
Alcohol Use	Yes	44	45.8
	No	52	54.2
Histological Grade	Well-differentiated	32	33.3
	Moderately differentiated	41	42.7
	Poorly differentiated	23	24
Clinical Stage	Stage I	18	18.8
	Stage II	25	26
	Stage III	29	30.2
	Stage IV	24	25
Lymph Node Metastasis	Present	39	40.6
	Absent	57	59.4

**Table 2:** Correlation of tumor marker expression with histological grade

**Marker Expression**	**Well-Differentiated (n=32)**	**Moderately Differentiated (n=41)**	**Poorly Differentiated (n=23)**	**p-value**
p53 Expression				0.003*
Negative	4 (12.5%)	2 (4.9%)	0 (0.0%)	
Low (10-50%)	9 (28.1%)	5 (12.2%)	1 (4.3%)	
High (>50%)	19 (59.4%)	34 (82.9%)	22 (95.7%)	
Ki-67 Index				<0.001*
Low (<25%)	18 (56.3%)	8 (19.5%)	1 (4.3%)	
Intermediate (25-50%)	11 (34.3%)	13 (31.7%)	2 (8.7%)	
High (>50%)	3 (9.4%)	20 (48.8%)	20 (87.0%)	
Mean±SD (%)	28.45±12.67	48.32±15.21	68.74±14.89	<0.001*
EGFR Expression				<0.001*
Negative	14 (43.8%)	10 (24.4%)	3 (13.0%)	
Low	11 (34.3%)	11 (26.8%)	3 (13.1%)	
High	7 (21.9%)	20 (48.8%)	17 (73.9%)	
*Statistically
significant (p<0.05)

**Table 3:** Association of tumor marker expression with lymph node metastasis

**Marker Expression**	**LN Metastasis Present (n=39)**	**LN Metastasis Absent (n=57)**	**OR (95% CI)**	**p-value**
p53 Overexpression (>50%)	36 (92.3%)	39 (68.4%)	5.54 (1.52-20.18)	0.006*
Ki-67 High (>50%)	28 (71.8%)	16 (28.1%)	6.50 (2.68-15.78)	<0.001*
EGFR High	26 (66.7%)	16 (28.1%)	5.13 (2.15-12.24)	<0.001*
Combined High Expression				<0.001*
All three markers high	21 (53.8%)	6 (10.5%)	9.92 (3.47-28.37)	
Two markers high	12 (30.8%)	18 (31.6%)	1.94 (0.76-4.96)	
One or no marker high	6 (15.4%)	33 (57.9%)	Reference	
Clinical Stage Association				
Stage I-II (n=43)	8 (20.5%)	35 (61.4%)	-	<0.001*
Stage III-IV (n=53)	31 (79.5%)	22 (38.6%)	-	
*Statistically
significant (p<0.05);
OR: Odds Ratio;
CI: Confidence Interval;
LN: Lymph Node
